# Epidemiological Survey on the Perception of Adverse Effects in Women Using Contraceptive Methods in Brazil

**DOI:** 10.1055/s-0041-1741410

**Published:** 2022-01-29

**Authors:** Gabriel Duque Pannain, Vivian de Oliveira Rodrigues Brum, Maria Mariana Andrade Abreu, Gabriela Barbosa Lima

**Affiliations:** 1 Department of Surgery, Section of Gynecology, Faculdade de Medicina, Universidade Federal de Juiz de Fora, Juiz de Fora, MG, Brazil

**Keywords:** contraception, hormonal contraception, drug-related side effects and adverse reactions, gynecology, epidemiology, contracepção, contracepção hormonal, efeitos colaterais e reações adversas relacionadas ao uso de medicamentos, ginecologia, epidemiologia

## Abstract

**Objective** The present study aimed to understand patient perception of the adverse effects of contraceptives to improve health care and adherence to treatment.

**Methods** An online questionnaire was available for women in Brazil to respond to assess their perception of adverse effects and their relationship with contraceptive methods.

**Results** Of all 536 women who responded, 346 (64.6%) reported current contraceptive use. One hundred and twenty-two (122–34.8%) women reported having already stopped using contraception because of the adverse effects. As for the contraceptive method used, the most frequent was the combined oral contraceptive (212–39.6%). When we calculated the relative risk for headache, there was a relative risk of 2.1282 (1.3425–3.3739; 95% CI), suggesting that the use of pills increases the risk of headache, as well as edema, in which a relative risk of 1.4435 (1.0177–2.0474; 95% CI) was observed. For low libido, the use of oral hormonal contraceptives was also shown to be a risk factor since its relative risk was 1.8805 (1.3527–2.6142; 95% CI). As for acne, the use of hormonal contraceptives proved to be a protective factor, with a relative risk of 0.3015 (0.1789–0.5082; 95% CI).

**Conclusion** The choice of a contraceptive method must always be individualized, and the patients must be equal participants in the process knowing the expected benefits and harms of each method and hormone, when present.

## Resumo

**Objetivo** Este estudo é destinado a entender a percepção de pacientes sobre os efeitos adversos dos métodos contraceptivos para aprimorar o atendimento médico e a aderência das mulheres ao tratamento.

**Métodos** Um questionário online foi disponibilizado para que mulheres no Brasil respondessem a fim de avaliar a sua percepção em relação aos efeitos adversos e a associação desses aos métodos contraceptivos.

**Resultados** Das 536 mulheres que responderam, 346 (64,5%) alegaram uso atual de método contraceptivo. Cento e vinte e duas (122–34,8%) mulheres disseram que já haviam parado o uso de métodos contraceptivos devido aos seus efeitos adversos. Quanto ao método contraceptivo em uso, o mais frequentemente utilizado foi o contraceptivo hormonal oral combinado (212–39,6%). Quando calculamos o risco relativo para cefaleia, foi encontrado um risco relativo de 2,1282 (1,3425–3,3739; 95% intervalo de confiança [IC]), sugerindo que o uso das pílulas aumenta o risco de ocorrência desse efeito adverso, bem como de edema, cujo risco relativo foi de 1,4435 (1,0177–2,0474; 95% IC). Em relação à redução da libido, o uso de contraceptivo hormonal oral combinado foi também considerado um fator de risco, pois seu risco relativo foi 1,8805 (1,3527–2,6142; 95% IC). No que se refere à acne, o uso de contraceptivos hormonais demonstrou ser um fator de proteção, com risco relativo de 0,3015 (0,1789–0,5082; 95% IC).

**Conclusão** A escolha de um método contraceptivo deve sempre ser individualizada, e as pacientes devem participar igualmente nesse processo sabendo dos benefícios e malefícios esperados de cada método e hormônio, quando presente.

## Introduction

Prevention of pregnancy can be achieved by inhibiting ovulation, fertilization, and/or implantation using hormonal or non-hormonal methods. Hormonal contraception can be accomplished through oral contraceptive pills, transdermal patches, vaginal rings, subcutaneous implants, intramuscular injections, or intrauterine devices. 1

In 2015, it was estimated that almost 650 million women worldwide used oral contraceptives. 2 However, despite its broad use, only a few use it, as it is prescribed by its gynecologist. 3 In a 2015 research, only 56% women reported being protected by a contraceptive method at the moment of their last vaginal intercourse. 4

Long-acting reversible contraception methods (LARCs) are known to be the most effective due to not being dependent on the active participation of the patient—as is the case with the pill—and because of its long-acting mechanism, whether through liberation of progestogens, as in hormonal intrauterine devices (IUDs) and intradermal implants, or induction of an inflammatory response to hinder the process of fecundation, as in the copper and silver IUDs. 5

However, women worldwide are more likely to use oral contraceptive pills or male condoms. 4 Contraceptive pills can be composed by two types of hormones, ethinyl estradiol or estradiol valerate paired with a form of progestogen, or by only one type, progestogen. 6

Even though both types of pills can inhibit follicle-stimulating hormone (FSH) and luteinizing hormone (LH) sufficiently to prevent ovulation, the combined pills have greater antigonadotrophic and ovulation-inhibition effects. The progestogen component is effective in blocking the midcycle rise in LH secretion, which inhibits ovulation. On the other hand, ethinyl estradiol is mainly used due to its beneficial effect of preventing irregular shedding of the endometrium. 7

In contraception, the most used hormones are estradiol, estradiol valerate, and ethinyl estradiol, as shown in Table 1 . The dosage of estrogen has been decreased over the years to reduce the risk of cardiovascular disorders. However, reducing the estrogen dose leads to a less favorable control of the menstrual bleeding. 8

**Table 1:** Estrogens, their origin, and their uses in gynecology

Estrogen	Origin	Contraception use	Therapy hormone use
Estrone	Natural	No	No
Estradiol	Natural	Oral	No
Estriol	Natural	No	Oral, topical vaginal
Estetrol	Natural	Oral (under study)	Oral (under study)
Ethinylestradiol	synthetic	Oral, vaginal ring, adhesive	No
Promestriene	synthetic	No	Topical vaginal
Estradiol Valerate	synthetic	Oral, monthly injectable	Oral

Progestogens are classically characterized according to their structural origins, and the main ones can be seen in Table 2 . They can bind to androgens, glucocorticoids, and mineralocorticoid receptors. According to its structure and activated receptors, each progestogen is associated with a greater or lesser frequency of adverse effects. 9

**Table 2:** Main progestogens used in gynecology and their effects

	Antiestrogenic	Antiandrogenic	Glucocorticoid	Antimineralocorticoid
Cyproterone	+	++	+	−
Chlormadinone	+	+	+	−
Dienogest	+-	+	−	−
Drospirenone	+	+	−	+
Gestodene	+	−	+	+
Levonorgestrel	+	−	−	−
Nomegestrol	+	+-	−	−

+ strong action; + - moderate action; - weak action

Contraceptive methods may cause other effects that are dependent on the type and dose of the estrogenic and progestogenic components. These effects are often the reason a particular method is chosen. Among them, the most notable are the improvement in the appearance of hirsutism and acne when ethinylestradiol is used in combination with antiandrogenic progestogen. 10 11

Worldwide, the most common reasons for the non-use of contraception and the discontinuation of the method are fear of side effects and health concerns. 11 12 However, data about this topic in Brazil are scarce.

Even in women with high awareness, statements such as “IUDs are an abortive method” and “amenorrhea is prejudicial to health” are extremely common. These myths and misconceptions must be addressed by the medical assistant. 13

The present study aimed to understand patient perception of the adverse effects of contraceptives to improve health care and adherence to treatment.

## Methods

The method of selection for the population of this study was convenience sampling. Since the number of women in reproductive age in our region is ∼ 43 million, we wanted a sample of at least 385 women.

Women of reproductive age over 18 from a city in Brazil who had already had contact with the local university hospital, either as a patient, doctor, academic, or employee and who were using or had used any type of contraceptive methods were invited to answer an electronic questionnaire through an online link. All women who had an online contact registered in our database ( *n* = 1,274) received an email with the invitation to answer an online questionnaire. Five hundred and thirty-six women (536) filled the online questionnaire, after signing the informed consent form.

The questionnaire was divided into three parts. The first part evaluated the patient's demographic data. The second performed a brief medical screening to assess if there were any comorbidities. The questions in the second part were intended to assess whether such symptoms existed prior to the use of the method, whether they were related to the menstrual cycle and whether they improved or worsened with the use of the contraceptive method. Finally, the last part analyzed the use of contraceptive methods and their correlation with adverse effects. It evaluated whether the patient currently uses contraception, what dosage, frequency and time of use, reason for discontinuing use, and it also questioned previous use of emergency contraception. So, the relationship between contraceptives and the main adverse effects reported by patients (headache, edema, mastalgia, and low sexual libido) could be evaluated.

To better understand the perception of the patients, all side effects (edema, headache, acne and mastalgia) were reported by self-diagnosis. No complaints were excluded, even if the women had hyperandrogenism or migraine, since we wanted to evaluate if they perceived an improvement or worsening of their symptoms after using the contraceptive method.

On the online questionnaire, the diagnosis of low libido was based on the deficiency or absence of sexual fantasies and desire for sexual activity or in cases in which the deficiency causes marked distress or interpersonal difficulty in more than half of sexual intercourse in the month. Having that in mind, women could mark that option if they thought it was well-suited to their complaints.

This project was approved by legal ethics committee under CAAE 34836620.2.0000.5133.

## Results

The mean age of the women who answered the questionnaire was 24.08 years, and the median of 23 years, with the highest age being a single woman aged 51 years, and the lowest fourteen women aged 18 years. The most reported profession was student (reported by 264 women, which corresponded to 49.3% of the total), followed by doctor (35 women—6.5%), teacher (11 women—2.1%), and lawyer (11 women—2.1%).

Eighty-nine (89—16.6%) of the women reported having some comorbidity, with the most frequent being asthma (present in 21—3.9%), followed by polycystic ovary syndrome (12—2.2%), and mood disorders (8—1.5%). Other reported comorbidities were hypothyroidism (6.7%), hyperthyroidism (1.1%), unspecified thrombophilia (1.1%), diabetes mellitus (2 women), systemic arterial hypertension (2.2%), migraine (3.3%), endometriosis (4.4).

Of all 536 women who participated in the study, 346 (64.6%) reported current use of a contraceptive method, 79 (14.7%) said they had already used it, but were no longer doing it, and 11 (2.1%) reported never having used contraception. In those who currently used it, the average use of the same contraceptive was 5.5 years, and the median was 5 years, with the lowest being 25 women who had not completed even 1 year of use (4.7%), and the highest being a woman who has used the same method for 25 years (0.2%). All those patients who had previously used contraceptives and discontinued their use (100%) were using combined oral contraceptives.

Three hundred and fifty-one (351—65.5%) patients reported having stopped using contraception for more than a month at some point. They reported the most frequent reason was the presence of side effects (122—34.8%), followed by not using it right (20—5.7%), no more need for contraception (19—5.4%), fear of frequent use of hormone (6—1.7%), and pregnancy (2—0.5%).

The most frequently used method was the combined oral contraceptive (212—39.6%), followed by the concomitant use of combined oral contraceptive and condom (93—17.4%), levonorgestrel-releasing intrauterine system (LRIS) (26—4.9), copper IUD (19—3.5), condom (18—3.4%), monthly injectable contraceptive (4—0.7%), contraceptive quarterly injectable (2—0.4%), implant (2–0.4%), and withdrawal (1—0.2%). In addition, 277 (51.7%) participants reported having used the morning-after pill at least once, even during the use of regular contraception.

Among women using hormonal pills, the most used combinations were 3 mg of drospirenone and 0.02 mg of ethinylestradiol (68 women—12.7%); 2 mg of cyproterone acetate combined with 0.035 mg of ethinylestradiol (63 patients—11.8%); 3 mg of drospirenone associated with 0.03 mg of ethinylestradiol (25 women—4.7%); 0.060 mg of gestodene with 0.015 mg of ethinylestradiol (17 women—3.2%); dienogest and estradiol valerate (13 women—2.4%); 0.075 mg of gestodene associated with 0.03 mg of ethinylestradiol (12 women—2.2%); 0.15 mg of levonorgestrel associated with 0.03 mg of ethinylestradiol (11 women—2.1%); and 2 mg chlormadinone acetate plus 0.03 mg ethinyl estradiol (10 women—1.9%). Other combinations mentioned were 0.075 mg of gestodene associated with 0.02 of ethinylestradiol (8 women—1.5%); 0.075 mcg of desogestrel (9 women—1.7%); 0.15 mcg of desogestrel associated with 0.020 of ethinylestradiol (7 women—1.3%); 2.5 mg of nomegestrol acetate associated with 1.5 mg of estradiol (3 women—0.6%); and 2 mg of chlormadinone acetate associated with 0.020 mg of ethinylestradiol.

As for side effects, 110 (20.5%) women reported headache using a contraceptive method, 72 women (13.4%) reported spotting. Among those with spotting, 22 (30.5%) reported feeling uncomfortable, but not enough to suspend the use of the method. On the other hand, 7 (9.7%) answered that they stopped using the pill due to the side effects. When evaluating which combination the patients were using, those ones most related to spotting were the combination of 0.02 mg ethinylestradiol to 3 mg drospirenone (15 patients); 0.03 mg ethinylestradiol with 0.075 mg gestodene (6 patients); 0.03 mg of ethinylestradiol combined with 3 mg of drospirenone (6 patients) ( Tables 3 , 4 , and 5 ).

**Table 3:** Percentage of adverse effects after using the pill and its composition

	0.03 mg ethinylestradiol + 0.075 mg gestodene	0.03 mg ethinylestradiol + 3 mg drospirenone	0.015 mg ethinylestradiol + 0.060 mg gestodene	0.035 mg ethinyl estradiol + 2 mg cyproterone acetate	0.030 mg ethinylestradiol + 2 mg chlormadinone acetate	0.020 mg ethinylestradiol + 3 mg drospirenone	Estradiol valerate + dienogest
Spotting	42% (6)	24% (6)	5.9% (1)	7.9% (5)	20% (2)	22% (15)	31% (4)
Headache	36% (5)	20% (5)	12% (2)	33% (21)	40% (4)	31% (21)	30% (4)
Acne	0	0	0	3.2% (2)	0	2.9% (2)	7.6% (1)
Mastalgia	29% (4)	16% (4)	18% (3)	19% (12)	20% (2)	24% (16)	7.6% (1)
Edema	21% (3)	20% (5)	41% (7)	43% (27)	40% (4)	31% (21)	23% (3)
Low libido	78% (11)	48% (12)	59% (10)	44% (28)	30% (3)	46% (31)	23% (3)
Total users	14	25	17	63	10	68	13

**Table 4:** Percentage of adverse effects in women who used a contraceptive method other than the pill

	IUD	LRIS	Condom	Monthly injectable	Quarterly injectable	Implant
Spotting	16% (3)	27% (7)	0	12.5% (1)	25% (1)	50% (1)
Headache	0	0	5,5% (1)	12.5% (1)	50% (2)	0
Acne	32% (6)	58% (15)	11% (2)	12.5% (1)	0	50% (1)
Mastalgia	5% (1)	15% (4)	33% (6)	0	25% (1)	0
Edema	26% (5)	19% (5)	22% (4)	25% (2)	0	50% (1)
Low libido	5% (1)	19% (5)	11% (2)	37.5% (3)	50% (2)	50% (1)
Total users	19	26	18	8	4	2

Abbreviations: IUD, intrauterine device; LRIS, levonorgestrel-releasing intrauterine system.

**Table 5:** Women who reported improvement in symptoms after using contraceptive pills

	0.03 mg ethinylestradiol + 0.075 mg gestodene	0.03 mg ethinylestradiol + 3 mg drospirenone	0.015 mg ethinylestradiol + 0.060 mg gestodene	0.035 mg ethinyl estradiol + 2 mg cyproterone acetate	0.030 mg ethinylestradiol + 2 mg chlormadinone acetate	0.020 mg ethinylestradiol + 3 mg drospirenone	Estradiol valerate + dienogest
Spotting	0	8% (2)	12% (2)	6.4% (4)	0	4.4% (3)	0
Headache	71% (10)	56% (14)	47% (8)	79% (50)	70% (7)	62% (42)	62% (8)
Acne	7.1% (1)	24% (6)	29% (5)	14% (9)	10% (1)	21% (14)	38% (5)
Mastalgia	0	12% (3)	0	11% (7)	20% (2)	18% (12)	31% (4)
Edema	0	4.0% (1)	0	1.6% (1)	0	3% (2)	0
Total users	14	25	17	63	10	68	13

When the relative risk was calculated regarding the use of hormonal contraceptives for spotting and mastalgia, there was no statistical significance at the 95% confidence level. However, when calculated for headache, there was a relative risk of 2.1282 (1.3425–3.3739; 95% CI), suggesting that the use of pills increases the risk of headache as well as edema, for which a relative risk of 1.4435 (1.0177–2.0474; 95% CI) was observed. For low libido, the use of oral hormonal contraceptives was also shown to be a risk factor since its relative risk was 1.8805 (1.3527–2.6142; 95% CI). As for acne, the use of hormonal contraceptives proved to be a protective factor, with a relative risk of 0.3015 (0.1789–0.5082; 95% CI).

As for contraceptive methods other than the pill, the only one that showed any statistically significant calculation was the LRIS, which proved to be a risk factor for acne, with a relative risk of 6.8077 (4.3107–10.7510; 95% CI). However, it is important to note that the calculations were hampered by the low number of patients reporting the use of such method.

## Discussion

Women are increasingly becoming the protagonist in choosing their contraceptive method. However, this is the first study in this region to evaluate the methods collateral effects according to each specific formulation. Not only that, but it is also one of the few studies in which patients could make a self-diagnosis, which helped us understand their feelings in relation to the contraceptive methods.

Despite using an online questionnaire and a convenience sample, our findings were very similar to those of the literature worldwide, such as the mean age of users as 25 years old, the contraceptive pills as the most used method, and the main reason for dissatisfaction and discontinuation use of a contraceptive method. 13 14 15 16

Although the pill is still the most used contraceptive method, the frequency of women using LARCs is increasing. Nevertheless, because of various myths and misconceptions this transition is happening at a slow pace in Brazil. One of the main reasons for this is the decrease in the use of hormonal methods and the increase in methods which are either hormone-free or have a lower hormone dosage, which can result in lower side effects. 17 18

In view of the cardiovascular risk caused by estrogen, vasculopathic patients, such as those with hypertension, diabetes, obesity, and dyslipidemia, should avoid the use of hormonal methods, which may explain why the prevalence of these comorbidities was so low in our study. 15 16

Unplanned pregnancies are more common in women who are not satisfied with the contraceptive method they are using. 19 In our study, whose results agree with those of the literature, the main reason for dissatisfaction with a contraceptive method was adverse effects, such as spotting, headache, and low libido. 20

While progesterone is the hormone ultimately responsible for preventing pregnancy, the estrogen component helps regulating menstrual bleeding. 16 However, in our study, the findings regarding irregular bleeding, such as spotting, were more prevalent in women who used lower doses of estrogen, such as 0.020 mg. And, despite the use of a higher dose, a relevant number of women using 0.030 mg of ethinylestradiol with drospirenone also reported spotting. This finding can indicate that the control of menstrual bleeding is not only dependent on estrogen dosage but also on the progestogen used to compose the pill. Due to its antiestrogenic effect, the progestogen may impact the effect of estrogen, which makes sense since drospirenone has a strong antiestrogenic action as has already been suggested by some studies. 21

Another important adverse effect related to the use of contraceptives is headache, as shown in our study, which can be caused mainly by using combined pills. 22 It seems that every primary headache—as tension, migraine, and trigeminal-autonomous—is more prevalent in women, because female hormones have a main role on migraine. 23

In our study, women who used hormonal methods reported more headache than those who did not used any hormonal method. Moreover, among those using hormonal methods, patients who used a higher estrogen dose seemed to have had more headaches than those who used less than 0.02 mg of ethynyl estradiol. However, the exact relationship between headache and hormone dosages remains unknown, and it should be a topic of future research. 24

Unlike headaches, acne is more prevalent in those who do not use a hormonal method. And, unlike headaches, this relationship is already fully understood. 25 Acne is more frequent in women with hyperandrogenism, since the androgens not only regulate lipogenesis in sebocytes but also influence inflammation in acne and convert progesterone in testosterone. 24 Therefore, for women with acne, the best methods are those with a great antiandrogenic effect, such as cyproterone, which was observed in our study.

Mastalgia in women under contraceptive therapy is controversial, and, as shown in our study, it does not appear to suffer the influence of hormones. 26 The truth is that the reason why mastalgia happens is still unknown, even in women who do not use hormonal methods. 27 Regarding edema, drospirenone derivatives have emerged as an important treatment. Due to their antimineralocorticoid effects, they stimulate diuresis. 28 However, we must keep in mind that it is difficult to assess edema, especially when the study is performed based on patient report, as was our case.

Finally, one of the main patient complaints in relation to hormonal contraceptives is low libido. It is also one of the main reasons for discontinuation of the use of hormones and for switching to other methods, such as IUDs. 29 As seen in our study, it appears that women have a greater decrease in libido when using progestogens with greater antiestrogenic effect, such as gestodene, and it is a minor complaint in patients using pills containing dienogest, which has a minor antiestrogenic effect.

Despite this, more studies are needed, as the present study did not obtain a representative sample of the population in Brazil, since the majority of its population were white women and those in the medical field. Not only that, but as the women completed the questionnaire at home, some questions could have been misunderstood.

## Conclusion

It is essential to understand the relationship of women with their contraceptive methods to be able to provide better medical care, since unplanned and unwanted pregnancies can often result from the inadequate use of contraceptive methods due to dissatisfaction with the method. We also must keep in mind that contraceptive methods have other effects beyond preventing fecundation. They can also have secondary beneficial effects, such as: prevention of acne and irregular bleeding and management of dysmenorrhea. All the effects must be taken into consideration when prescribing any type of method. Moreover, the choice of a contraceptive method must always be individualized, and the patients must be equal participants in the process. They must be aware of the possible adverse effects of each method and must be willing to try more than once to find the ideal one.
